# Lost in the Wards: A Retrospective Study of Long-Term Psychiatric Hospitalization in a Tertiary Care Institute

**DOI:** 10.1192/j.eurpsy.2026.10775

**Published:** 2026-07-31

**Authors:** S. Khairwal, P. Gehlawat, S. Singh, D. Kumar

**Affiliations:** Psychiatry, Institute of human behavior and allied sciences, Delhi, India

## Abstract

**Introduction:**

Long-term psychiatric hospitalization persists in low- and middle-income countries despite global trends toward community-based care (Thornicroft & Tansella, Br J Psychiatry 2013;202:246-248). In India, lack of rehabilitation facilities and family abandonment are key contributors (Wig & Murthy, Indian J Psychiatry 2015;57:158-161; Kumar et al., Psychiatry Res 2021;299:114080).

**Objectives:**

Objectives:

1. To describe the sociodemographic and clinical profile of patients admitted for more than three months in a tertiary care psychiatric hospital.

2. To identify the clinical, familial, and systemic barriers contributing to prolonged hospitalization.

**Methods:**

This was a retrospective chart review conducted at the Institute of Human Behaviour and Allied Sciences (IHBAS), Delhi. Hospital records of all patients admitted for more than three months between January 2022 and December 2024 were reviewed. A total of 30 patients met the inclusion criteria. Data were extracted on sociodemographic details (age, sex, education, marital status, employment), clinical diagnosis, comorbidities, treatment history, family involvement, and systemic issues contributing to prolonged hospitalization. Data were analyzed descriptively. Ethical clearance was obtained from the Institutional Ethics Committee.

**Results:**

The study included 30 patients, the majority of whom were middle-aged men. Most patients were diagnosed with schizophrenia, followed by mood disorders and substance use disorders. Comorbid intellectual disability and personality traits were observed in several cases. A large proportion of patients were unemployed and came from low socioeconomic backgrounds. Family abandonment and lack of social support were identified in over half the cases. Systemic barriers such as absence of halfway homes and community rehabilitation centers contributed significantly to prolonged stay.

**Conclusions:**

Long-term psychiatric hospitalization remains a pressing issue in tertiary care institutes in India. The major drivers include treatment resistance, comorbidities, family abandonment, and lack of systemic rehabilitation options. Addressing these barriers requires coordinated efforts at clinical, social, and policy levels. There is an urgent need to establish halfway homes and community-based rehabilitation centers to ensure continuity of care and reduce unnecessary prolonged admissions.

**Disclosure of Interest:**

None Declared

